# The Effect of Lamb Vaccination Against Echinococcosis on the Scale of Livestock Farming in Northwestern China

**DOI:** 10.1002/vms3.70273

**Published:** 2025-03-03

**Authors:** Bingxin Liu, Qihui Chen, Jinshan Cai, Jing Li, Yumei Liu

**Affiliations:** ^1^ College of Economics & Management China Agricultural University Beijing P.R. China; ^2^ Beijing Food Safety Policy & Strategy Research Base China Agricultural University Beijing P.R. China; ^3^ Veterinary Public Health Department Qinghai Center for Animal Disease Prevention and Control Qinghai P.R. China

**Keywords:** China, echinococcosis, farming scale, households, lamb vaccination, staggered difference‐in‐differences method

## Abstract

Echinococcosis (also known as ‘hydatidosis’ or ‘hydatid disease’) is a severe zoonotic disease that poses threats to human and animal health and significantly impacts livestock production, which may lead to declines in both the quantity and quality of animal products and result in economic losses. Lamb vaccination against echinococcosis is considered an effective method for controlling the transmission of echinococcosis in the intermediate host—sheep, thereby disrupting the disease's transmission chain, and thus a crucial strategy for preventing and controlling echinococcosis. Based on field survey data on 581 households collected from 11 counties of China's Qinghai Province, this study evaluated the impact of lamb vaccination against echinococcosis on livestock farming scale using a staggered difference‐in‐differences method. The results show that on average, households that received lamb vaccination from their county as part of the ‘Action Plan for Echinococcosis Prevention and Control in Qinghai Province (2016–2020)’ had a livestock farming scale of 52.09 heads (or 36%) higher than those that did not receive lamb vaccination. This effect likely works through reductions in cattle and sheep mortality and culling numbers. A simple accounting analysis suggested that the increase in livestock farming scale can translate into a profit of 26,500 yuan (≈3938 US dollars at 2022 prices), although this impact has a short‐term lag and is limited in duration. While these findings may not apply to all livestock households in China, they suggest that developing area‐specific lamb vaccination programs, increasing lamb vaccination rates, and ensuring timely vaccinations are crucial for enhancing livestock farming scale in echinococcosis endemic areas.

## Graphical abstract

Lamb vaccination against echinococcosis significantly boosted the livestock farming scale by reducing cattle and sheep mortality and culling numbers in Qinghai.

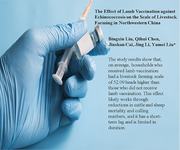

## 1 Introduction

Echinococcosis (also known as ‘hydatidosis’ or ‘hydatid disease’), a severe zoonotic disease with a global distribution, is caused by *Echinococcus granulosus*, a member of the Cestoda class and the Taeniidae family (Banda et al. 2013; Zhang and Xiao 2020). Hydatid cysts can parasitize cattle and sheep's livers and lungs, typically on the surface or inside of the parasitized organ, undermining the health of these animals and thus the quantity and quality of livestock products, ultimately causing significant productivity and economic losses in the livestock sector (Pereira et al. 2022). Echinococcosis can also infect humans through the consumption of improperly cooked meat and organs from contaminated animals (Jibat et al. 2008; Komba et al. 2012; Swai and Schoonman 2012), which may, in turn, lead to high morbidity and mortality rates in humans (Al‐Hindi et al. 2023; Lahmar et al. 2004; Nakao et al. 2013; Yibar et al. 2015).

Over time, more and more countries have recognized not only the veterinary significance but also the public health and economic importance of preventing and controlling echinococcosis in both animal and human populations (Schantz et al. 1995). Since Iceland implemented the first‐ever echinococcosis control program worldwide in 1863, many previously highly endemic countries, such as New Zealand and Tasmania, have launched similar programs, significantly reducing the echinococcosis incidence in these countries (Craig and Larrieu 2006; Craig et al. 2017). Various measures have been adopted in echinococcosis prevention and control programs in the past few decades, the most common ones including visceral apprehension, slaughterhouse development, dog population control, and awareness promotion (Interministériel de lutte contre l'Hydatidose/Echinococcose 2007; Saadi et al. 2021; Yang et al. 2022). More recently, trials of the EG95 vaccine in several countries, such as Argentina and Australia, have demonstrated its effectiveness in controlling echinococcosis (Lightowlers et al. 1999). Further research has found respectable echinococcosis control effects even in remote areas where only half of the lambs can be vaccinated (Larrieu et al. 2019).

China provides a recent case for assessing the effectiveness of lamb vaccination in echinococcosis control and prevention. Compared with many other countries, China has a relatively high prevalence of echinococcosis, partly because of the massive pastoral areas it is endowed with. As of 2021, there were a total of 30,421 echinococcosis‐endemic villages, with 26,773 recorded cases of echinococcosis patients across the country (Kui et al. 2023). Among all provinces in China, Qinghai has the highest infection rates and infection intensities in both livestock and humans (Liu et al. 2017).^1^ Despite recent declines in echinococcosis infection rates in cattle and sheep (Cai et al. 2013),^2^ preventing and controlling echinococcosis remains a critical issue in Qinghai. To win the battle against echinococcosis, the Qinghai Provincial Government issued the ‘Action Plan for Echinococcosis Prevention and Control in Qinghai Province (2016–2020)’ (hereafter, the ‘Action Plan’) in 2015, with lamb vaccination against echinococcosis as a major component. The primary procedure adopted in Qinghai was to administer three subcutaneous injections of 1 mL each in the neck of the lamb, with the subunit vaccine produced by the Chongqing AULEON Biologicals Co. The initial vaccination was given to newborn lambs at around 8 weeks of age, followed by a second vaccination 1 month later. A third vaccination was given after 12 months, with a 7‐day observation period after each vaccination.

Several studies have evaluated the performance of the Action Plan regarding its overall effectiveness and the effectiveness of its lamb‐vaccination component. The results, in general, demonstrate the success of the Action Plan in controlling and preventing echinococcosis. Not only has the Action Plan achieved veterinary success, significantly reducing human morbidity rates, terminal host infection rates, and intermediate host small rodent infection rates in Qinghai (Liu et al. 2017; Zhao et al. 2020), but it has also made great progress in the public health domain, greatly improving technical personnel's prevention and control knowledge and increased public awareness; considerable economic gains were also generated (Cai et al. 2023; Kan et al. 2017).

However, knowledge gaps remain. In particular, few have examined the impact of lamb vaccination (against echinococcosis) on the scale of livestock farming, an essential indicator of the development and strength of a country's livestock sector. Given the relatively small‐scale livestock farming in many developing countries, a higher level of specialization in animal husbandry and intensified management can be achieved by properly expanding the farming scale; as a result, economies of scale can be better realized (Bava et al. 2014; Schmalzried and Fallon 2007). Yet, as one of the most severe zoonotic diseases, echinococcosis not only undermines farmers’ confidence in livestock production but may also create consumer resistance to livestock products, leading to a decline in the market size and, ultimately, a shrinkage of the livestock sector. Given the crucial role lamb vaccination plays in interrupting the transmission of echinococcosis, credible evidence on the desirable effects of lamb vaccination on livestock farming scale could help restore farmers’ confidence, stabilize market demand, and generate economies of scale in animal husbandry, eventually ensuring the livestock industry's sustainable development. In this regard, how lamb vaccination against echinococcosis may impact livestock farming scale is a question of both academic interest and policy relevance.

To provide an answer, the present study intends to rigorously evaluate the effect of lamb vaccination against echinococcosis on the livestock farming scale in Qinghai.^3^ Specifically, we exploit temporal and regional variations in the implementation of the Action Plan across Qinghai's counties to develop a staggered difference‐in‐differences (DID) model and apply it to analyze a panel dataset on 581 livestock households collected from 11 counties in Qinghai. The results show that despite a certain lag in the short term, lamb vaccination against echinococcosis significantly raised the livestock farming scale in Qinghai (by 52.09 heads per household). This effect is primarily realized through the reduction of mortality and culling numbers of cattle and sheep.

This study adds to the veterinary literature on echinococcosis prevention and control by rigorously evaluating the effects of lamb vaccination against echinococcosis on livestock farming scale, an understudied indicator of livestock development, in China, thereby enriching existing research on echinococcosis control policy in developing countries. The findings may inform livestock households about potential tools for echinococcosis control and provide an essential reference for the government to consider policy options conducive to echinococcosis prevention and control.

## 2 Materials and Methods

### 2.1 Study Area

**FIGURE 1:**
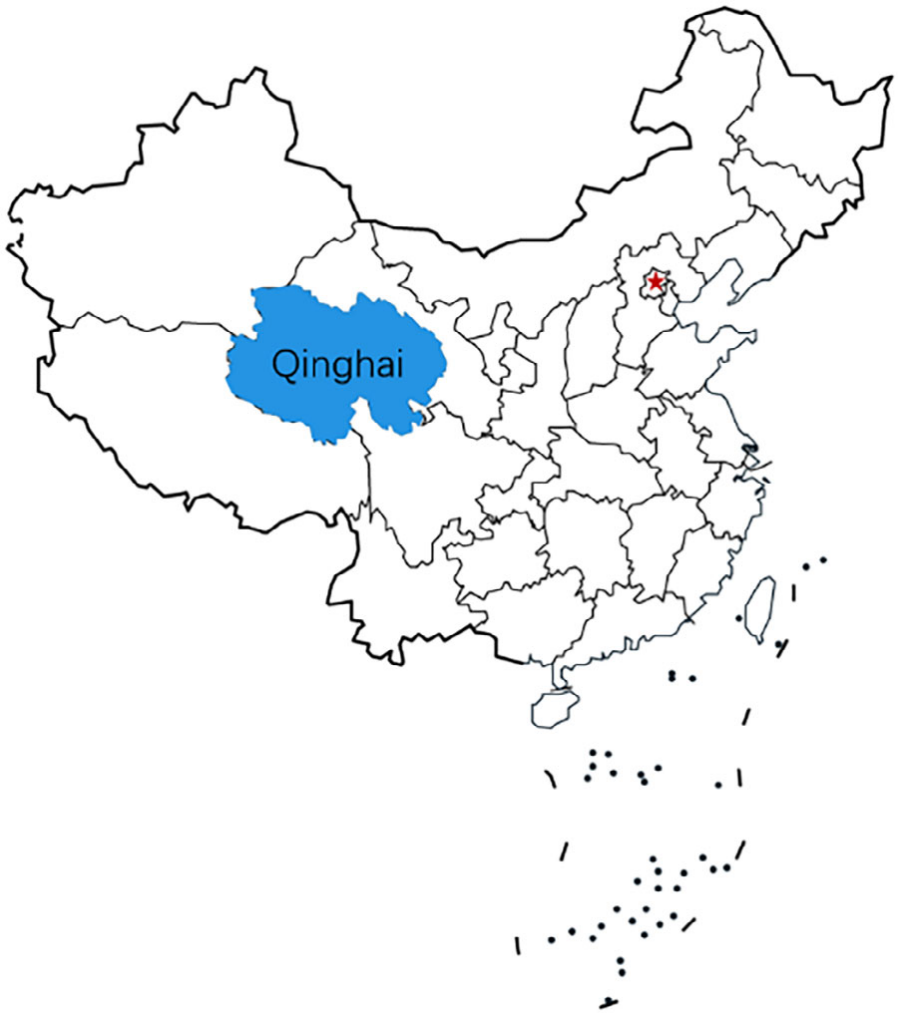
Geographic location map of Qinghai in China. *Source*: Standard Map Service System.

The study area of this study, Qinghai Province, is located in Northwestern China (Figure 1), deriving its name from Qinghai Lake, China's largest inland saltwater lake. The majority of Qinghai's landscape is situated on the Qinghai‐Tibet Plateau (*Qingzang Gaoyuan*), with an average elevation of 3500 m above sea level. Mainly because of its high altitude, the climate in Qinghai is characterized by low humidity, temperature, and oxygen levels. About 60% of Qinghai's total land area, approximately 722,300 km^2^ (the fourth largest nationwide), is covered by grassland, of which 92% can be used for livestock farming. The region also enjoys abundant sunlight for forage crop growth, with average annual sunshine hours of 2336–3341 and an average total radiation of 5860–7400 MJ/m^2^; direct radiation accounts for over 60% of the total radiation.^4^ These unique geographical and climatic conditions provide suitable conditions for animal husbandry, making Qinghai one of China's five major animal husbandry bases. Nevertheless, large‐scale livestock farming also renders Qinghai a high‐risk area for echinococcosis.

### 2.2 Data

Our analytical dataset consists of both official statistics and field survey data. Official county‐level statistics, provided by the Qinghai Province Animal Disease Prevention and Control Center (ADPCC), cover 42 counties in eight prefectures (including Xining, Haidong, Haibei, Huangnan, Hainan, Guoluo, Yushu, and Haixi) from 2016 to 2020.

Field survey data were collected through face‐to‐face interviews with livestock farming households in 11 counties in the eight aforementioned prefectures. In October 2021, the research team conducted pilot field visits in Qinghai Province to assess the effect of lamb vaccination against echinococcosis on echinococcosis prevention and control and interviewed representative households. Based on the results of pilot field visits, a formal survey questionnaire was designed and then refined after discussions with local experts from Qinghai ADPCC to ensure its validity and suitability for our study. The formal data collection took place from December 2021 to April 2022, based on a stratified random sampling plan.

The stratified random sampling plan we adopted involves several key steps. First, based on the classification criteria concerning echinococcosis infection levels provided in the Action Plan, we identified 32 level‐1 counties, seven level‐2 counties, and three level‐4 counties in Qinghai.^5^ Second, based on recommendations from experts at Qinghai ADPCC, we randomly selected eight level‐1 counties (Haiyan, Qilian, Huangyuan, Yushu, Guinan, Maduo, Henan, and Delingha), two level‐2 counties (Geermu and Huzhu), and one level‐4 county (the Dachaidan Administrative Committee, a county‐level administrative unit). Finally, based on the results of the sample size calculation (with reference to the studies by Bonett 2002, and Schulz and Grimes 2005), we randomly selected about 55 households per county,^6^ including three types of livestock households: cattle‐raising, sheep‐raising, and cattle‐sheep mixed‐raising.

The stratified random sampling plan intends to ensure the study sample's representativeness and reliability. However, owing to variations in livestock breeding conditions across Qinghai's counties, especially the difficulty in reaching households at high altitudes, the sampling plan could not be strictly followed in some areas. For example, in Yushu, Henan, Da Chaidan, and Delingha, the number of survey samples was smaller than 55 households. To mitigate the loss of statistical power caused by the reduced sample size in these four counties, we increased the sample size of households interviewed in some other counties (Huzhu, Haiyan, Qilian, Huangyuan, and Golmud) subject to logistics considerations. Ultimately, a sample with 581 valid observations was collected, with a sample loss rate of only 3.2%, which still allows us to detect desirable effects. The total sample includes 157 sheep‐raising households, 189 cattle‐raising households, and 235 ‘mixed’ households that raise both cattle and sheep (Table 1).

**TABLE 1:** Characteristics of sampled counties.

City	County	Sheep‐raising households	Cattle‐raising households	Cattle‐sheep mixed‐raising households	Total
Haidong	Huzhu	31	27	31	89
Haibei	Haiyan	28	24	26	78
	Qilian	0	0	84	84
Xining	Huangyuan	27	28	26	81
Yushu	Yushu	0	40	0	40
Hainan	Guinan	23	9	20	52
Guoluo	Maduo	19	20	16	55
Huangnan	Henan	3	5	3	11
Golmud	Golmud	22	29	29	80
Haixi	Da Chai Dan	4	0	0	4
	Delingha	0	7	0	7

*Source*: Author's calculation based on survey data on livestock households in Qinghai.

The data collected contain information on sample households’ livestock situation from 2015 to 2021, including the scale of sheep and cattle farming, farm endowments (number of labourers and grassland area), grassland characteristics (whether under government regulation), quality of life, living environment, and personal characteristics of the household head (age, education level, farming experience, whether he/she served as a village cadre, religious beliefs, risk preferences,^7^ and whether he/she had contracted echinococcosis).

Table 2 presents summary statistics for the sample. On average, sampled households had 235.63 heads of sheep and cattle, managing 71.77 hundred acres of grassland with 4.37 labourers. Lamb vaccination against echinococcosis was received by 38% of the households. The majority (98%) of household heads in the sample were male, with a relatively low average educational level (7.09 years). Only 4% of household heads in the sample had contracted echinococcosis before.

**TABLE 2:** Descriptive statistics of variables used in the analysis, all household‐by‐year observations (N = 581).

Variables	Definitions	Mean (“Yes”%)	Standard deviation	Min.	Max.
Outcome variables (Y)					
Sheep and cattle farming scale	Heads	235.63	292.10	0	4550
The natural logarithm of the farming scale		4.54	1.76	0	8.42
Household farming scale with 2 years of pre‐ and post‐vaccination observation records	Heads	365.06	343.41	0	4550
Sheep‐raising households and mixed households’ farming scale	Heads	316.83	322.14	0	4550
Sheep‐raising households’ farming scale	Heads	365.21	345.71	0	4550
Cattle‐raising households’ farming scale	Heads	67.24	71.07	0	406
Key explanatory variable (D)					
Lamb vaccination	0 = No; 1 = Yes	38%		0	1
Control variables (X)					
Endowments for livestock production					
Grassland area	Hundred acres	71.77	289.08	0	3600
Number of labourers	Persons/household	4.37	1.59	0	11
Characteristics of household head					
Gender	0 = Female;1 = Male	98%		0	1
Age	Years	43.87	9.96	20	77
Village cadres	0 = No; 1 = Yes	10%		0	1
Farming experience	0 = No; 1 = Yes	88%		0	1
Suffered from echinococcosis	0 = No; 1 = Yes	4%		0	1
Religious beliefs	0 = No; 1 = Yes	58%		0	1
Education level	Years	7.09	4.58	0	15
Risk preference of household head (reference: risk averse)					
Risk neutrality	0 = No; 1 = Yes	17%		0	1
Risk loving	0 = No; 1 = Yes	17%		0	1
Grassland characteristics					
Grassland subject to governmental regulation	0 = No; 1 = Yes	2%		0	1
Mediating variable (M)					
The natural logarithm of cattle and sheep mortality and culling numbers		2.69	1.43	0.18	7.76

*Source*: Author's calculation based on survey data on livestock households in Qinghai.

### 2.3 Statistical Methods

According to the Action Plan, lamb vaccination against echinococcosis was implemented for sheep breeders in the 11 aforementioned project counties, but with clear temporal variations. In 2016, Yushu, Maduo, Henan, and Guinan counties, where echinococcosis was relatively more prevalent, implemented lamb vaccination, followed by Qilian and Haiyan in 2019 and Golmud, Delingha, and Dachaidan in 2020. Huangyuan and Huzhu did not implement lamb vaccination until 2022. Apart from this temporal pattern, an administrative feature is also helpful for the purpose of our study: lamb vaccination against echinococcosis was not voluntary at the household level. Rather, if a county was selected as a project county for implementing lamb vaccination, all livestock households within that county would receive lamb vaccination. The temporal variation of local implementation of lamb vaccination against echinococcosis, along with the abovementioned administrative feature, provides a valuable opportunity for applying a staggered DID model, the current workhorse in impact evaluations based on observational data (Baker et al. 2022; Beck et al. 2010; Carrieri et al. 2020; Freyaldenhoven et al. 2019; Haaga et al. 2024), to evaluate the effects of the lamb vaccination against echinococcosis on livestock farming scale in Qinghai.

#### 2.3.1 Assessing the Overall Impact of Lamb Vaccination

Formally, we construct the following statistical model:

(1) $$Y_{it}=\alpha +\beta D_{it}+\delta X_{it}+\gamma_{i}+\eta_{t}+\varepsilon_{it},$$

where subscripts *i* (= 1, 2, …, 581) and *t* (= 2015, 2016,…, 2021) refer to sampled households and years, respectively. The outcome variable of interest, $Y_{it}$, represents six different variables, depending on the specific context: the total scale of sheep and cattle farming, the natural logarithm of the total farming scale, household farming scale with 2 years of pre‐ and post‐vaccination observation records, the farming scale of sheep‐raising households and mixed households, that of sheep‐raising households, and that of cattle‐raising households (Table 2). The key explanatory variable, $D_{it}$, is an indicator whose value equals one if household *i* had received lamb vaccination by year *t* (‘treated’) and zero (‘untreated’) otherwise. $X_{it}$ is a set of time‐varying covariates, including households’ livestock endowments and grassland conditions, as well as the basic characteristics and risk preferences of the household head discussed above (Table 2). $\gamma_{i}$ and $\eta_{t}$ represent, respectively, household and year fixed effects. $\varepsilon_{it}$ is the disturbance term. In essence, Equation (1) is a so‐called ‘two‐way fixed effects’ model.

In this setup, the parameter β captures the impact of lamb vaccination against echinococcosis on the scale of livestock farming. Intuitively, β measures whether the outcome variable *Y* increased faster during the intervention period (2015–2021) for households who received lamb vaccination (the ‘treatment’ group) compared to those who did not receive lamb vaccination (the untreated ‘control’ group). A positive and statistically significant estimate of β will provide initial evidence that the implementation of lamb vaccination indeed raised the livestock farming scale in Qinghai.

#### 2.3.2 Testing the Parallel‐Trend Assumption and Dynamic Effects

The key assumption for the benchmark DID model (1) to yield unbiased estimates of β is the so‐called ‘parallel trend’ assumption. That is, in the absence of lamb vaccination against echinococcosis, the time trends of (the mean of) outcome *Y* for the ‘treatment’ group (those who had received lamb vaccination by *t*, $D_{it}=1$) and the ‘control’ group (those who had not received lamb vaccination during the study period, $D_{it}=0$) should be similar (‘parallel’). If this assumption holds, the impact of lamb vaccination can be assessed by the difference in post‐implementation time trends of *Y*.

To incorporate tests for the parallel‐trend assumption and capture possible dynamic effects of lamb vaccination against echinococcosis, we follow the standard practice in the literature (Baker et al. 2022; Beck et al. 2010; Freyaldenhoven et al. 2019) and include a set of year dummies in the benchmark model (1) to trace out the year‐by‐year effects of lamb vaccination against echinococcosis on livestock farming scale:

(2) $$Y_{it}=\alpha +\sum_{j=0}^{k}\beta_{j}D_{it}^{j}+\sum_{j=-1}^{-f}\beta_{j}D_{it}^{j}+\delta X_{it}+\gamma_{i}+\eta_{t}+\varepsilon_{it},\;\cdot k\leq 4,f\leq 4,$$

where $D_{it}^{j}$ ’s are dummy variables indicating the time periods relative to the period of lamb vaccination for household *i*, $D_{it}^{0}$. More specifically, the $D_{it}^{j}$ ’s are defined as follows. First, let $s_{i}$ denote the specific year in which a household *i* received lamb vaccination. Following Luo and Qi (2021), we set the year immediately before lamb vaccination as the baseline year. Further, define $j=t-s_{i}$ ​, and let $D_{it}^{j}=1$ if year *j* is the *t*th year post‐vaccination for household *i*, and $D_{it}^{j}=0$ otherwise. In this setup, the coefficient $\beta_{j}$ captures the impact of lamb vaccination in time period *j*. Because very few sampled households have more than four periods of observations before or after lamb vaccination, we set $D_{it}^{-4}$ equal to one for all years prior to the fourth pre‐vaccination year. Similarly, we set $D_{it}^{4}$ equal to one for all years after the fourth post‐vaccination year.

The parallel‐trend assumption implies that when *j* < 0, the coefficients of $D_{it}^{j}$ ’s should not be statistically significantly different from zero, meaning that there were no significant differences in the pre‐vaccination trends of outcome *Y* between the treatment and the control groups. Additionally, by estimating changes in livestock farming scale over different pre‐ and post‐vaccination periods using the set of time‐period dummies $D_{it}^{j}$, model (2) can capture the dynamic effects brought about by lamb vaccination against echinococcosis on outcome *Y*.

## 3 Results

### 3.1 Descriptive Results

Table 3 presents the average scale of livestock farming for different survey years by sampled households’ treatment status: with lamb vaccination (panel A) or without (panel B). The rollout nature of the intervention is clearly revealed: no sampled households had received lamb vaccination against echinococcosis by 2015, but from 2017 on, the number of households that had received lamb vaccination against echinococcosis increased sharply. Note that the table provides preliminary evidence of lamb vaccination's positive impact on livestock farming scale in Qinghai: the average livestock farming scale for households that had received lamb vaccination against echinococcosis increased significantly from 2017 to 2021 (Column 1); in contrast, that of those households who had not received lamb vaccination against echinococcosis decreased notably during the same period (Column 3). Note also that this evidence is only suggestive, as the above contrasts were observed without netting out the influence of potential confounding factors. More reliable evidence should be obtained based on models that can properly control for confounding factors, to which we now turn.

**TABLE 3:** Number of livestock farming households with or without lamb vaccination against echinococcosis and their average farming scale, 2015–2021 (N = 581).

	Lamb vaccination implemented		Lamb vaccination not implemented	
Year	(1) Scale of sheep and cattle (heads/farm)	(2) Number of households	(3) Scale of sheep and cattle (heads/farm)	(4) Number of households
2015	0	0	213.61	581
2017	164.82	158	247.27	423
2019	280.33	320	194.87	261
2021	317.51	411	128.26	170

*Source*: Author's calculation based on survey data on livestock households in Qinghai.

### 3.2 Difference‐in‐Differences Estimates

#### 3.2.1 Benchmark Results

Table 4 reports the main results of estimating a number of versions of the benchmark staggered DID model (1). Column 1 presents the simplest model without control variables, showing that on average, households that received lamb vaccination against echinococcosis had a livestock farming scale of 40.37 heads larger than those that did not receive lamb vaccination against echinococcosis; the difference is statistically significant at the 1% level. Column (2) further includes a series of control variables discussed in Section 2.2, and the average difference between the two types of households increased to 52.09 heads and is, again, significant at the 1% level. These DID estimates confirm that lamb vaccination significantly boosted the livestock farming scale in Qinghai.

**TABLE 4:** Results of staggered DID estimation.

	(1)	(2)	(3)	(4)	(5)	(6)	(7)	(8)	(9)
	Benchmark estimates				Robustness checks				Mechanism analysis
Variables	Livestock farming scale	Livestock farming scale	Log (livestock farming scale)	Log (livestock farming scale)	Households with 2 years of pre‐ and post‐lamb‐vaccination obs.	Cattle‐raising households excluded	Sheep‐raising households only	Cattle‐raising households only	Log (cattle and sheep mortality and culling numbers)
Lamb vaccination	40.37***	52.09***	0.27***	0.36***	125.11**	49.84***	83.75**	−5.94	−0.38***
	(10.58)	(15.07)	(0.07)	(0.10)	(50.69)	(14.56)	(34.18)	(3.97)	(0.13)
Labor force		16.48*		0.32***	51.12	13.11*	10.76	1.38	−0.16**
		(8.46)		(0.07)	(57.08)	(7.60)	(8.28)	(3.63)	(0.08)
Age		−0.41		0.01	0.12	−0.62	−3.62**	0.44***	−0.29***
		(0.97)		(0.01)	(1.33)	(0.97)	(1.66)	(0.14)	(0.03)
Farming experience		−19.47		0.47**	−71.55	−16.18	37.54	−0.38	−0.51**
		(17.88)		(0.23)	(53.19)	(16.94)	(37.34)	(4.16)	(0.24)
Village cadres		−34.69		0.25**	−120.05	−21.98	74.53	−7.09	−0.09
		(52.61)		(0.10)	(116.75)	(47.45)	(62.35)	(8.03)	(0.18)
Suffered from echinococcosis		626.74***		3.74***	615.19***	607.40***	−130.63	−25.87	−3.36***
		(41.00)		(0.22)	(232.80)	(40.57)	(84.62)	(42.14)	(0.29)
Has religious beliefs		−77.81		−0.39	317.88**	−95.52	170.59**	−72.77***	−3.42***
		(74.47)		(1.55)	(157.16)	(72.78)	(76.07)	(10.37)	(0.13)
Education level		2.30		−0.02	43.62*	1.56	−166.59	4.16***	−0.39***
		(4.81)		(0.02)	(24.37)	(4.15)	(102.01)	(1.27)	(0.03)
Risk neutrality		315.31**		3.12**	366.75*	296.46**	1629.39*	0.29	1.64***
		(127.91)		(1.56)	(213.33)	(122.47)	(984.43)	(10.76)	(0.30)
Risk appetite		253.56***		3.29***	−52.93	270.57***	−133.56	−22.03	−1.52***
		(66.71)		(0.28)	(153.75)	(60.97)	(92.38)	(25.47)	(0.40)
Grassland area		−0.27		−0.00	−13.50	−0.25	−3.78	1.93	0.00
		(0.23)		(0.00)	(8.49)	(0.22)	(2.49)	(1.31)	(0.00)
Grassland subject to governmental regulation		−14.75		0.29	−127.99**	−20.77	3.56	8.02	0.09
		(34.72)		(0.54)	(63.87)	(34.09)	(76.64)	(5.34)	(0.76)
Year fixed effects	Yes	Yes	Yes	Yes	Yes	Yes	Yes	Yes	Yes
Household fixed effects	Yes	Yes	Yes	Yes	Yes	Yes	Yes	Yes	Yes
Constant	242.49***	39.77	5.44***	0.62	−472.98	74.48	1700.90*	47.90**	21.55***
	(52.24)	(112.63)	(0.20)	(1.67)	(418.96)	(107.74)	(922.71)	(22.99)	(1.45)
*R* ^2^	0.83	0.82	0.83	0.83	0.79	0.82	0.73	0.95	0.86
N	2324	1248	2324	1248	372	1284	336	432	389

*Note*: Robustness standard errors are given in parentheses. ^∗∗∗^,^∗∗^, and ^∗^ indicate statistical significance at the 1%, 5%, and 10% levels, respectively.

*Source*: Author's estimation based on survey data on livestock households in Qinghai.

To assess the economic benefits associated with lamb vaccination against echinococcosis, assume all 52 heads of increased livestock due to lamb vaccination are sheep. On the revenue side, since the average carcass weight of a sheep in Qinghai is ∼17.3 kg, with a price of about 70 yuan/kg,^8^ the value of these 52 heads of additional sheep is ∼63,000 yuan (≈9396 US dollars in 2022 value). On the cost side, note that because lamb vaccination against echinococcosis was fully funded by the government, households did not bear the cost of vaccines. Given the livestock farming cost, ∼700 yuan per sheep, the value of the 52 additional sheep due to lamb vaccination implies an estimated profit of about 26,500 yuan (≈3938 US dollars in 2022 value).

Further using the natural logarithm of household farming scale as the outcome measure, Columns (3) and (4) of Table 4 reveal that lamb vaccination against echinococcosis increased not only households’ farming scale but also its growth rate: households with lamb vaccination experienced a 27% (Column 3) to 35% (Column 4) increase in farming scale relative to the scale prior to lamb vaccination than those without lamb vaccination. This impact is, again, statistically significant at the 1% level.

#### 3.2.2 Parallel‐Trend Tests and Dynamic Effects Estimation

**FIGURE 2:**
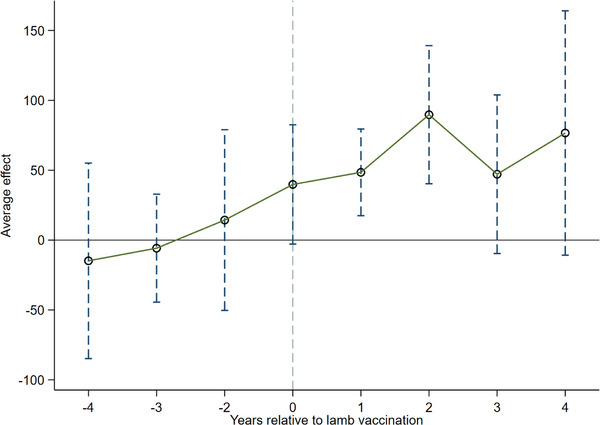
Parallel trend test and dynamic effects of lamb vaccination against echinococcosis. *Note*: Figure 2 corresponds to Table 5, Column 1. *Source*: Author's estimation based on survey data on livestock households in Qinghai.

The validity of the DID estimates presented above hinges on the plausibility of the parallel‐trend assumption discussed in Section 2.3. Table 5 presents the results of testing this assumption, along with estimates of the dynamic effects of lamb vaccination against echinococcosis (Equation 2). Reassuringly, the estimated treatment‐control differences in livestock farming scale in the fourth ($D_{it}^{-4}$), third ($D_{it}^{-3}$), and second ($D_{it}^{-2}$) pre‐vaccination years do not significantly differ from the difference in the baseline year ($D_{it}^{-1}$, the reference category), regardless of whether control variables are included in the model. These results, visualized in Figure 2, reveal no significant differences between the treatment and control groups in livestock farming scale in the absence of lamb vaccination against echinococcosis, lending strong support to the parallel‐trend assumption and the validity of our DID estimates.

**TABLE 5:** Results of testing the parallel‐trend assumption and estimating dynamic effects of lamb vaccination against echinococcosis.

Variables	(1) Livestock farming scale	(2) Livestock farming scale
D^−4^	−14.82	−28.34
	(42.50)	(68.78)
D^−3^	−5.78	−6.95
	(23.47)	(40.45)
D^−2^	14.34	37.17
	(39.29)	(63.25)
Current (D0)	39.82	53.24
	(25.93)	(41.37)
D^1^	48.49**	72.13**
	(18.84)	(28.68)
D^2^	89.72***	135.15***
	(30.02)	(47.50)
D^3^	47.12	71.12
	(34.49)	(53.53)
D^4^	76.63	96.07
	(53.12)	(84.62)
Control variables	No	Yes
Year fixed effects	Yes	Yes
Household fixed effects	Yes	Yes
Constant	244.96***	25.73
	(72.83)	(147.23)
*R* ^2^	0.83	0.82
N	2324	1248

*Note*: ^∗∗∗^,^∗∗^, and ^∗^ indicate statistical significance at the 1%, 5%, and 10% levels, respectively.

*Source*: Author's estimation based on survey data on livestock households in Qinghai.

In contrast, the significantly positive coefficients of the 1‐year ($D_{it}^{1}$) and 2‐year ($D_{it}^{2}$) post‐vaccination dummies suggest notable treatment‐control differences in livestock farming scale after lamb vaccination. Note that the coefficient of the year‐of‐vaccination dummy ($D_{it}^{0}$) is not statistically significant, suggesting a short‐term lag in the effect of lamb vaccination against echinococcosis. Note also that the impacts for the third ($D_{it}^{3}$) and fourth ($D_{it}^{4}$) years post‐vaccination are no longer statistically significant, suggesting that the effect of lamb vaccination against echinococcosis faded after a few years of implementation.

#### 3.2.3 Robustness Checks

To further strengthen our findings, we performed additional robustness checks to rule out several potential threats to the validity of our DID estimates. First, not all ‘treated’ households have data for 4 years before and after lamb vaccination against echinococcosis. Given the dynamic patterns revealed in Table 5, variations in the number of pre‐ and post‐intervention periods may have contaminated our estimates by mixing short‐term and long‐term impacts. To address this issue, we kept data only for households with records for 2 years before and 2 years after lamb vaccination and re‐estimated Equation (1). Consistent with our benchmark estimates (Table 4, Columns 1–2), the new estimates (Table 4, Column 5) confirm that lamb vaccination against echinococcosis significantly boosted households’ livestock farming scale.

Second, presumably, lamb vaccination against echinococcosis can only directly affect the farming scale of sheep, but not other animals. Given rural households’ limited resources, there exists a certain degree of substitutability between sheep and cattle raising, which can affect households’ livestock farming structure and, thus, the scale of cattle farming. As such, the above analysis pooling cattle‐ and sheep‐raising households may have attenuated the estimated impact of lamb vaccination. To check this possibility, we re‐estimated the benchmark model (1), excluding pure cattle‐raising households (Table 4, Column 6) and both cattle‐raising and mixed households (Table 4, column 7) from the sample. The results, again, reveal positive and statistically significant impacts of lamb vaccination against echinococcosis on the livestock farming scale—the estimated effect for pure sheep‐raising households (Table 4, Column 7) is much larger than that for all three types of households combined (Table 4, Column 2).

The third concern comes from the fact that the Action Plan consists of more than a lamb vaccination component; other components include dog management and deworming, slaughter management, and personnel training (Cai et al. 2023). Even though our DID design exploited temporal and regional variations tied explicitly to the implementation of lamb vaccination to identify its impact, the estimated effects might still be confounded with other components of the Action Plan, especially when the implementation of some other components overlapped with lamb vaccination. Yet, recognizing that lamb vaccination against echinococcosis was specifically targeted at sheep while other components (say, slaughter management) could impact other animals as well, we may devise a placebo test to rule out the influence of other components, focusing on households with no sheep. If our benchmark DID model indeed captured the impact of other components, the placebo DID model is expected to yield a statistically significant ‘lamb vaccination’ impact on pure cattle‐raising households. The result of this placebo test, reported in Column (8) of Table 4, turns out to be reassuring. The estimated effect of lamb vaccination against echinococcosis on cattle farming scale is small and statistically insignificant, suggesting our benchmark DID model isolates the impact of lamb vaccination against echinococcosis from other components reasonably well.

Finally, to provide corroborative evidence of our findings above, we examine a specific mechanism underlying this impact—sheep and cattle mortality and culling numbers. Naturally, to help increase livestock farming scale, lamb vaccination against echinococcosis should be able to reduce sheep and cattle mortality and culling numbers first. It has been found that lamb vaccination against echinococcosis can reduce the incidence of echinococcosis among sheep, with the average antibody qualification post‐vaccination rate reaching 65% (Zhao et al. 2020). The detection of immune antibodies in vaccinated lambs also confirmed that the vaccine administered provides adequate immune protection against sheep echinococcus granulosus. Lamb vaccination against echinococcosis is also highly effective in reducing the transmission of tapeworm infections within sheep (Amarir et al. 2021). The resultant improvement in overall health status may reduce mortality and culling numbers due to tapeworm infections, thereby raising livestock farming scale. As reported in Column (9) of Table 4, households with lamb vaccination experienced a 38% reduction in cattle and sheep mortality and culling numbers, an effect that is significant at the 1% level, providing corroborative evidence of the effect of lamb vaccination against echinococcosis on livestock farming scale.

Furthermore, the secondary data we obtained also validate the effectiveness of lamb vaccination in the prevention and control of echinococcosis. We obtained data on the positive rate of sheep echinococcosis in Yushu, Guinan, and Haiyan Counties from the Qinghai ADPCC for the years 2016 to 2020. The positive rates of echinococcosis in sheep in Yushu and Guinan were 11.25% and 16.67%, respectively, in 2016. By 2017, the positive rates of echinococcosis in sheep in the two counties had decreased to 2.1% and 12%, respectively. Haiyan County began vaccinating lambs in 2019, and by 2020, the positive rate of echinococcosis tested in sheep had decreased from 4.99% to 2%. One year after the vaccination of lambs, the positive rates in Yushu, Guinan, and Haiyan decreased by 9.15%, 4.47%, and 2.99%, respectively, confirming the positive effect of lamb vaccination against echinococcosis and proving the reliability of our mechanism (Table 6).

**TABLE 6:** Detection positivity rate of echinococcosis in sheep.

County	Year	Number of sheep tested for echinococcosis (heads)	Number of sheep testing positive for echinococcosis (heads)	Positive rate of sheep (%)
Yushu	2016	160	18	11.25
	2017	143	3	2.10
	2018	160	2	1.25
	2019	150	1	0.67
	2020	151	0	0
Guinan	2016	150	25	16.67
	2017	300	36	12.00
	2018	200	13	6.50
	2019	250	9	3.60
	2020	250	6	2.40
Haiyan	2016	173	28	16.18
	2017	289	45	15.57
	2018	300	15	5.00
	2019	361	18	4.99
	2020	401	8	2.00

*Source*: Data provided by the Qinghai Province Animal Disease Prevention and Control Center.

## 4 Discussion

### 4.1 Interpretation of Findings

Echinococcosis is causing significant losses to livestock farming in China (Ma et al. 2021; Wang et al. 2013). Although lamb vaccination against echinococcosis has been proven to be an effective measure for controlling the spread of this disease in other countries (Amarir et al. 2021; Heath et al. 2012; Larrieu and Zanini 2012), it has not yet been widely adopted across the massive pastoral regions in China. This study examines the role lamb vaccination plays in echinococcosis control and management, focusing on its effect on households’ livestock farming scale. To the best of our knowledge, this study is the first rigorous evaluation of the impact of lamb vaccination against echinococcosis on livestock farming scale in China. Based on panel data collected from 581 livestock households in 11 counties in Qinghai, our findings provide robust evidence that lamb vaccination against echinococcosis has a significantly positive impact on livestock farming scale—households that received lamb vaccination raised 52–125 more heads of livestock than their counterparts without lamb vaccination, depending on the specific analytical sample used, and the increased animals due to lamb vaccination can translate into a revenue of 63,000–151,000 yuan (≈9361–22,437 US dollars at 2022 prices). After deducting relevant costs, the net profit is 26,500–63,800 yuan (≈3938–9480 US dollars at 2022 prices), suggesting a return on investment of ∼73%.

Three important findings are worth discussing. First, although both sheep and cattle serve as intermediate hosts for echinococcosis parasites’ eggs, lamb vaccination against echinococcosis directly targeted sheep, resulting in an increase of 83.75 heads in the flock size for sheep‐raising households. The impact on mixed farming households is more complicated. On the one hand, lamb vaccination against echinococcosis improves the health of sheep, thereby reducing the opportunity cost of sheep farming and making it more cost‐effective. This might induce households to raise more sheep and fewer cattle in the short run. This suggests a short‐run substitution relationship between cattle and sheep raising, which explains why the impact of lamb vaccination against echinococcosis on livestock farming scale became greater after excluding cattle households and mixed households (Table 4, Column 7). On the other hand, as the number of sheep increases over time, market prices for sheep and mutton may reduce, leading households to allocate more resources to raising cattle, whose prices are relatively high. Moreover, the improvement in grazing conditions can also reduce the infection rate of bovine echinococcosis, thereby indirectly increasing the scale of cattle farming—a positive spillover effect. However, the insignificant impact of lamb vaccination against echinococcosis for cattle‐raising households (Table 4, Column 7) does not support this spillover hypothesis. Although the EG95 vaccine has also proven effective for cattle as well (Heath et al. 2012), given budget constraints, the Chinese government currently vaccinates lambs but not cattle. The lack of spillover effect we found necessitates the extension of vaccination programs to other species, including cattle, in the future.

Secondly, the impact of lamb vaccination against echinococcosis on livestock scale exhibits a short‐term lag effect and is limited in duration, with the most significant impact occurring in the second year post‐implementation. The major reason is that it takes about a year after lamb vaccination for the number of mature cysts to decrease significantly before reductions in the infection rate of livestock with echinococcosis can take effect (Torgerson 2003). Since the vaccine can only prevent new infections and cannot stop or eliminate existing cysts parasitizing the sheep, the infected lambs will only show a significant reduction in cysts ∼1 year after vaccination, leading to the observed delay in impact. Over the subsequent years, the reduction in infection pressure gradually increases. Once older sheep have been removed (sold or eliminated with non‐hazardous treatments), reinfection will only occur in dogs and cattle without preventive measures, thus limiting the impact on livestock farming scale. This implies that the government should regularly administer vaccines to lambs in the future to ensure the beneficial effects are sustainable.

Finally, we found lamb vaccination against echinococcosis improved livestock farming scale by reducing the mortality and culling numbers of cattle and sheep. However, the improvement in livestock farming scale is premised on the effective prevention and control of echinococcosis through lamb vaccination, which reduces the mortality and culling number of cattle and sheep caused by the disease. We obtained supporting evidence from second‐hand sources that lamb vaccination can effectively prevent and control echinococcosis, and 1 year after lamb vaccination, the positive rates of echinococcosis in sheep in Yushu, Guinan, and Haiyan decreased by 9.15%, 4.47%, and 2.99%, respectively (Table 4, Column 9). In addition, numerous studies have demonstrated the effectiveness of lamb vaccination in the prevention and control of echinococcosis. For instance, Lightowlers et al. (1999) conducted a randomized controlled trial using the EG95 recombinant vaccine in Australia and Argentina, which had the same active ingredient as the lamb vaccine used in Qinghai. They found that 86% of the vaccinated sheep had no active echinococcal cysts 1 year after the vaccination. Compared to the unvaccinated control group, vaccination reduced the number of active cysts by 99.3%. Larrieu et al. (2013) found that in the third year post‐vaccination, the positive rate of sheep echinococcosis on farms had dropped to 7.8% in Argentina. Pilot field trial results conducted by Larrieu et al. (2019) in Argentina indicated that before the introduction of the EG95 vaccine against echinococcosis, 56.3% of 6‐year‐old sheep were infected with echinococcosis at autopsy, and 84.2% of farms had infected sheep. After the introduction of the EG95 vaccine, only 21.6% of sheep over 6 years old were found to be infected at autopsy, and 20.2% of farms were found to have infected sheep. A similar experiment was conducted in Qinghai. The necropsy results showed that the infection rate and intensity of echinococcosis in the unvaccinated control group of sheep were 86.67% and 3.43, respectively. In contrast, the vaccinated treatment group of sheep showed an infection rate and intensity of 45.76% and 0.86, respectively, resulting in an immunological protection rate (cyst reduction rate) of 69.38% (Kan et al. 2017). These corroborative findings confirm the key role lamb vaccination plays in controlling echinococcosis and provide indirect evidence supporting the reliability of our findings.

### 4.2 Limitations

Despite these informative findings, this study is not without limitations. First, as this study is based on data from a single province, Qinghai, its findings may not be generalizable to other regions in China. Nevertheless, our data are the most recent and comprehensively available in China. Thus, our study still provides the first available set of evidence of the effectiveness of lamb vaccination in echinococcosis control in China. Second, our data were collected as a biennial panel. While the staggered DID approach can control for the impact of time‐invariant factors, it is unable to capture concurrent changes in the intervention years. Furthermore, due to the complexity of the livestock farming environment in Qinghai, we cannot entirely eliminate the influence of other types of livestock farming, such as cattle farming, even though we have excluded cattle‐raising households in our robustness analysis reported in Section 4.

## 5 Conclusion

Using a survey of 581 livestock households in Qinghai Province, this study investigated the impact of lamb vaccination against echinococcosis on the scale of livestock farming. Based on a staggered DID method, the study found that households that received lamb vaccination increased the scale of livestock farming by 52.09 heads, equivalent to a 36% growth, compared to those that did not receive lamb vaccination. Furthermore, an analysis of the underlying mechanism reveals that lamb vaccination against echinococcosis enhances livestock farming scale by reducing mortality and culling numbers of livestock. These findings indicate that the implementation of lamb vaccination against echinococcosis can effectively increase livestock households’ farming scale, which may translate into significant economic benefits. However, it is important to note that the impact has a short‐term lag effect and is limited in duration, necessitating regular vaccinations to maintain its desirable effects.

## Author Contributions

**Bingxin Liu**: writing – review and editing, writing – original draft, visualization, validation, methodology, software, formal analysis, conceptualization. **Qihui Chen**: writing – review and editing, supervision, methodology. **Jinshan Cai**: supervision, resources, project administration, funding acquisition, conceptualization. **Jing Li**: supervision, resources, project administration, investigation. **Yumei Liu**: writing – review and editing, methodology, data curation, investigation, funding acquisition.

## Ethics Statement

The authors have nothing to report.

## Conflicts of Interest

The authors declare no conflicts of interest.

### Peer Review

The peer review history for this article is available at https://www.webofscience.com/api/gateway/wos/peer‐review/10.1002/vms3.70273.

## Data Availability

None of the data were deposited in an official repository. The data that support the study findings are available from authors upon request.

## Endnotes

1 Infection intensity is defined as the total number of parasites (adults, larvae, eggs, or cysts) divided by the total number of infected livestock animals.

2 In 2000, Cai et al. (2013) conducted a survey to assess the prevalence of *Echinococcus granulosus* infection in cattle and sheep in Qinghai. The results revealed that the infection rate of echinococcosis in sheep was 53.14% (infection rate = total number of infected livestock/number of examinations × 100%) with an average infection intensity of 4.41 in counties such as Chengduo, Gande, Zeku, Delingha, and Guinan. Similarly, the infection rate of echinococcosis in cattle was 60.36% with an average infection intensity of 5.59. A decade later, the average infection rate of echinococcosis in sheep in Guinan, Chengduo, Xining, and Delingha counties decreased to 39.02%, accompanied by an average infection intensity of 2.4. Likewise, the average infection rate of echinococcosis in cattle decreased to 33.12% with an average infection intensity of 1.3 in 2010.

3 While lamb vaccination is a key measure for echinococcosis control and prevention, it is not the only option. Other measures include dog management and deworming, slaughter management and training, and public education (Cai et al., 2023). Since our data do not contain sufficient information for us to rigorously evaluate the effectiveness of other measures, we focus on the effect of lamb vaccination in echinococcosis control and management in this study.

4 Source: http://www.qinghai.gov.cn/dmqh/system/2020/10/10/010368324.shtml.

5 Qinghai's 42 counties were categorized into four echinococcosis infection levels: 32 were categorized as echinococcosis infection level‐1 (with a human infection rate ≥ 1% or a dog infection rate ≥ 5%), seven as level‐2 (with a human infection rate of 0.1%–1% or a dog infection rate of 1%–5%), and three as level‐4 (with a human infection rate < 0.05% or a dog infection rate < 0.1%). In 2012, there were no ‘level‐3’ counties in Qinghai.

6 Sample size calculation formula:$\;N=\frac{Z_{\alpha /2}^{2}\times \sigma^{2}}{\delta^{2}}$, where $Z_{\alpha }$ is the critical value corresponding to a given significance level *α* for a *t* distribution, to which our impact estimate *β* divided by its standard error se(*β*) converges in a large sample. In our study, we set *α* = 0.1 (for a two‐sided hypothesis test), which yields a $\;Z_{\alpha /2}$ of 1.64; σ represents the population standard deviation of livestock farming scale, which we have set to be 3 based on preliminary survey results. δ is an allowable error, which is set at 0.2. Given these parameter values, the above formula yields a sample size needed of *N* = 600, covering 11 counties with about 55 households per county. Under the assumptions made above, this sample size allows us to detect a statistically significant change in livestock scale due to lamb vaccination at the 10% significance level.

7 To assess the degree of risk that household heads are willing to undertake in the process of livestock farming, we measure their risk preferences. This was done based on their answers to the following question: Among the following three options, which one would you choose for winning a prize? (1) Directly receiving 2000 yuan; (2) 50% chance of winning 5000 yuan; (3) 20% chance of winning 15,000 yuan. Option 1 corresponds to ‘risk aversion’, option 2 corresponds to ‘risk neutrality’, and option 3 corresponds to ‘risk loving’.

8 Source: http://www.moa.gov.cn/xw/qg/201411/t20141126_4253965.htm.
